# Cyber–Physical Perception Interface for Co-Simulation Applications

**DOI:** 10.3390/s24196412

**Published:** 2024-10-03

**Authors:** Teodora Mîndra, Ana Magdalena Anghel

**Affiliations:** Faculty of Automatic Control and Computers, National University of Science and Technology POLITEHNICA Bucharest, 313 Splaiul Independentei, 060042 Bucharest, Romania; mindrateodora@gmail.com

**Keywords:** cyber–physical, co-simulation, interface

## Abstract

Co-simulation can bring improvements to the development of cyber–physical perceptive systems (CPPS) in critical fields, allowing uninterrupted system operation and flexibility to use both real-time sensor data and non-real-time data. This paper proposes a co-simulation approach that integrates physical systems and communication systems, including both hardware and software components. This study demonstrates how systems of different natures with discrete or continuous events can be simulated using three methods: time stepped, global event driven, and variable stepped. Through two case studies from the medical and energy fields, CPPS and co-simulation reveal their importance for the future by improving precision and efficiency, which leads to more accurate diagnoses and personalized treatments in the medical field and increases the stability of energy networks.

## 1. Introduction

Cyber–physical systems (CPS) have gained significant attention as part of the technology core that allows the achievement of Industry 4.0 objectives and the association with artificial intelligence (AI) techniques, with applications in the area of networked control systems (NCS) where logistic support is provided by communication networks that up to this point have been used in monitoring and control systems of multiple industrial fields for connecting or interconnecting controllers with field devices.

NCS performance can be evaluated by comparing context identification methodologies with the basis of the control method based on a series of communication protocols. The evaluation can be complicated by meticulous processes, and relief can come from numerical simulation tools, which can analyze the state of communication and control. Despite the benefit of reducing time and complexity, there are currently few integrated platforms for accurate simulation for CPS, but there are tools for modeling and testing communication networks or physical systems. Thus, a concept was proposed that combines the two simulators for physical and communication systems, called co-simulation.

By combining multiple software simulators and hardware emulators, a co-simulator offers a unified representation of the entire system edge. This allows for simultaneous testing across all integrated components.

The aim of the present work is to propose a solution that ensures a cyber–physical co-simulation (CPCS) that is versatile enough to be applied in as many applications as possible from different fields. 

In this way, cyber–physical co-simulation forms the management components of a platform called the monitoring and data processing platform (MDPP), including mature simulators capable of concrete results.

The contribution of this work is the extension of the co-simulation procedure, which usually referred only to the coupling of several software simulators in a specialized framework implemented on the platform, for example, digital twin (DT) type, to the integration of informational data provided by the environment, possibly from simulators and hardware emulators (based on HIL simulation—Hardware In the Loop). This extension is made through an interface structure called the Cyber–Physical Perception Interface (CPPI). The main role of the CPPI is to ensure that the two simulators run simultaneously in a cooperative manner, especially in terms of their time management.

The present work supports the applications of sensing devices in various industries. In the first case study, processing components, such as Programmable Logic Controllers, were used alongside industry-specific sensors, such as battery temperature sensors and irradiance sensors, to measure solar radiation on photovoltaic panels to estimate energy production. Also, current and voltage sensors were integrated, and the basis of the parameters introduced in the co-simulation process is described in the following sections of this paper.

This technical framework serves as a foundation for future expansion of medical research, especially in the context of co-simulation applied to improving the benefits of an esophageal prosthesis used in cancer treatments. In a future case study, parameters obtained from biosensors, which monitor blood glucose, tumor markers, and inflammation, and also sensors involved in swallowing detection and activation of artificial muscles to facilitate the swallowing process, will be analyzed.

This work makes an important contribution to the field of sensors and co-simulation, highlighting the potential of advanced sensing technologies in multiple fields, from renewable energy to medical applications.

## 2. Related Work

Relevant research in the field of cyber–physical systems is rich and in constant development, but to point out the most recent achievements and trends, we have chosen only two recent surveys that also address simulation issues [1,2], the last of which is focused on the Smart Grid (SG) field, which is one of the two main topics addressed in this paper. In the co-simulation area, there are also numerous references, many of them from a period before the introduction of the CPS concept. Among the first works that mention the need for a specialized interface for CPS, we noted [3] for emphasizing the perceptive aspect of the interface. Recent contributions add to the basic co-simulation procedures some new ones involving artificial intelligence techniques, especially through machine learning [4], but this line of evolution was not followed in our work. Instead, we prefer the approach of using a digital twin as a simulation environment, and this idea is also described in a very recent synthesis paper [5], which underlines the advantages of using the open-source digital twin framework, OpenTwins, in co-simulation applications.

The rest of the work mentioned in this section is related to applications that refer to the co-simulation of cyber–physical systems, grouped into two categories depending on the thematic area represented in our work through two case studies: Cyber–Physical Power Systems (CPPS) and Medical Cyber–Physical Systems (MCPS).

Regarding research on co-simulation methods for CPPS, we first mention a recent comprehensive survey [6] that provides a concept map of common concepts found in the context of a Smart Grid co-simulation domain covering several relevant aspects, such as applications, platform selections, synchronization aspects, architectures, and benefits. Another survey that cannot be neglected, although it appeared 5 years ago, is detailed in [7]. It presents 26 SG co-simulation frameworks divided into several types based on principles such as topics, simulation tools, and synchronization methods and having correlations between the essential structures, challenges in research, and future work for analysis. Another important work that is relevant to the solution provided when solving co-simulation is the integration of two conventional simulators, one for the physical systems and the other for communication networks [8]. Additionally, based on the analysis of the solutions offered for the management of the simulation time, we chose for our work the proposed method of synchronizing the two co-simulators. 

Another work that presents the problem found between the communication network and the physical energy network is [9], which presents the framework on which the CPPS is formed and presents several modeling methods that are focused on the incidence characteristic matrix that provides a series of information related to theories of hybrid system. This paper also presents a comparison of the two distinct categories of simulation from the point of view of the dynamics of the studied processes, non-real-time co-simulation vs. real-time co-simulation, which helped us choose the simulation methods used in this work. A particular problem, that of coordinating companion simulators when using a distributed co-simulation protocol (DCP), was solved in [10] using a compliant implementation of an interoperable interface between a 3D environment, a physical simulator, and a co-simulation platform. The same problem is discussed in [11], which offers a detailed study of the advantages and disadvantages when using a DCP. 

Other specific solutions that solve certain particular aspects of CPPS management can be found in the following:

- A novel co-simulation platform for performance evaluation of an SG [12];
- Co-simulation is used to power CPS in risk prediction [13];
- Co-simulation is provided by an interlinked three-layer distributed control framework for the coordinated operation of multiple distributed energy resources in microgrids [14];
- Co-simulation and communication architecture for digital twin and virtual reality software [15].

The specialized literature also proposes a work for the field of MCPS and medical device systems (MDs) [16] by analyzing the results of a questionnaire about the infrastructure of the systems with an emphasis on increasing the efficiency and medical services. Similarly, [17] presents the co-similarity approach as a tool for representing subsystems in software and hardware systems, the objective being to describe an independent system health analysis mechanism that operators can use. Human–machine interfaces (HMIs) in CPS were co-simulated in [18] with models based on aspects of critical situations. A Functional Mockup Interface (FMI) is used in complex systems, and co-simulation is performed by integrating components and several simulators as [19], interconnecting the software part and the hardware part and thus connecting an HMI for representation.

In another approach [20], one can consider the problem of co-simulation, which is applied as a phantom simulator, to emulate the functionality of an organ, introducing computational human phantoms (CHPs) for automatic and precise creation based on the particular characteristics of the patient as the mathematical basis for the representation of human anatomy. 

Similarly, ref. [21] presents a methodology for the design and development of a repeatable experimental simulation platform that allows the personalization factor to be applied to a different scenario for testing.

Other specific solutions that solve certain particular aspects of MCPS management can be found in the following:

- Novel co-simulation platform where the validation of smart medical CPS can be realized [22];
- Co-simulation allows us to detect faults more effectively and efficiently than the baseline techniques [23];
- It is recommended to study situations that are challengingly dynamic and thus require efficient control and communication simulators [24];
- Considering previous research, this paper proposes a solution that provides a flexible and adaptable cyber–physical co-simulation to various applications, including from completely different domains. The authors have already analyzed the applicability of the solution in a case study, and as a future direction, they plan to develop a new application for CPCS.

## 3. Co-Simulation Aspects

In this session, we present different ways of approaching co-simulation processes, with the desire to have solutions for as many applications as possible, even if the specific case studies referred to in the final part of the paper refer to the coordination of cooperation between two simulators in order to keep the generality. In practical applications, there are many risks of failure in connecting several simulators; thus, several solutions have been proposed through which co-simulators reduce the challenges encountered, such as the correct choice of architecture and data synchronization (non-real and real time). This last proposed feature, which is used to control the simulation time, has the advantage that it will perform simulation actions faster than real systems. The nature of the system must also be chosen and be between discrete and continuous in order to establish the rate of change in the variables that will be used. The combination of the two types of systems, continuous time with discrete events, is still found in communication networks, having results [25] that are not limited to the nature of a single system through hybrid co-simulation.

The present paper proposes traditional methods for time synchronization in co-simulators, where physical systems are continuous-time dynamic systems represented by differential equations. In this way, the objective of the co-simulation is to respond to the set of differential equations through procedures that start with the discretization in sampling time. Most of the time, establishing the nature of the communication systems is based on the analysis of the network topology, the transmitted data packets, and the communication protocols, and that is why methods based on discrete events are chosen for communication systems, starting from a set of events and the moment they were recorded, with the new data being added. 

In order to solve the challenge of simulating two different systems, three methods based on precision (global event driven), execution time stepped, and implementation difficulties (variable-stepped method) were proposed in this paper.

### 3.1. Time-Stepped Method

This approach has the simplest procedure, and synchronization is performed and is periodically proportional to the sampling step of the physical system. For each synchronization moment, the simulators take data and the state; afterwards, the simulation continues independently until the next sampling step. 

**Figure 1:**
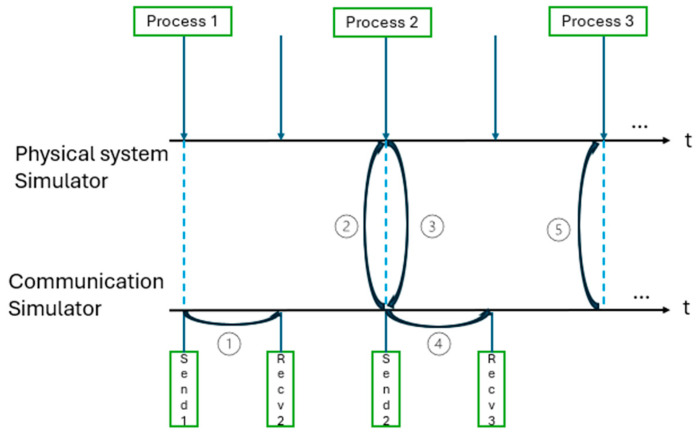
Demonstrative illustration of the time-stepped method (after [6]).

In Figure 1, timelines of the two simulators [6] are presented; the simulator running with the periodic sampling times is indicated by the vertical arrows and is shown at the top of the diagram for the physical system and is used for the communication system with lower sampling times, and stopping when events occur is indicated by the squares at the bottom of the figure.

### 3.2. Global Event-Driven Method

Another method is the one that offers flexibility by adapting according to the events that take place during the simulations, thus determining the synchronization times of the systems that change at any sampling moment between the simulators.

**Figure 2:**
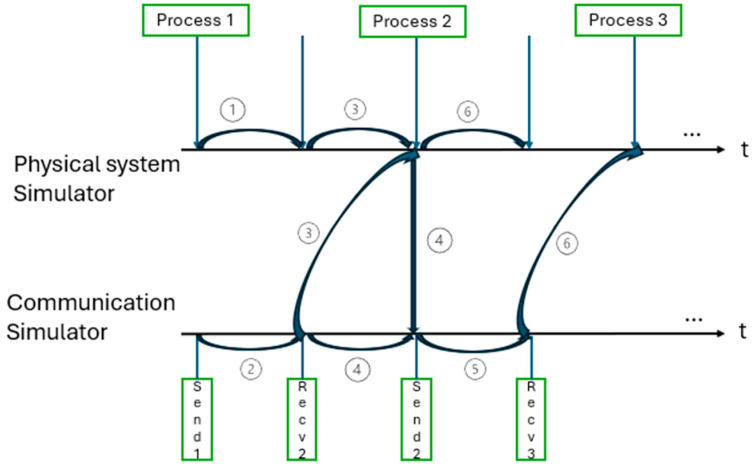
Demonstrative illustration of the global event-driven method (after [6]).

Starting in Figure 1, Figure 2 presents a particular case conceived in which the data packets are transmitted because the arrival times of the messages are different, arriving earlier for the physical system and requiring the introduction of an event handler to advance the synchronization clocks for the next meeting where synchronization takes place.

### 3.3. Variable-Stepped Method

This method is based on global events, which transform the simulator into a time-stepped delayer and are adapted to the communication events that occur between periodic sampling times. The delayer causes the physical system simulator to stop at the time of the event, and any received message will take place immediately. 

**Figure 3:**
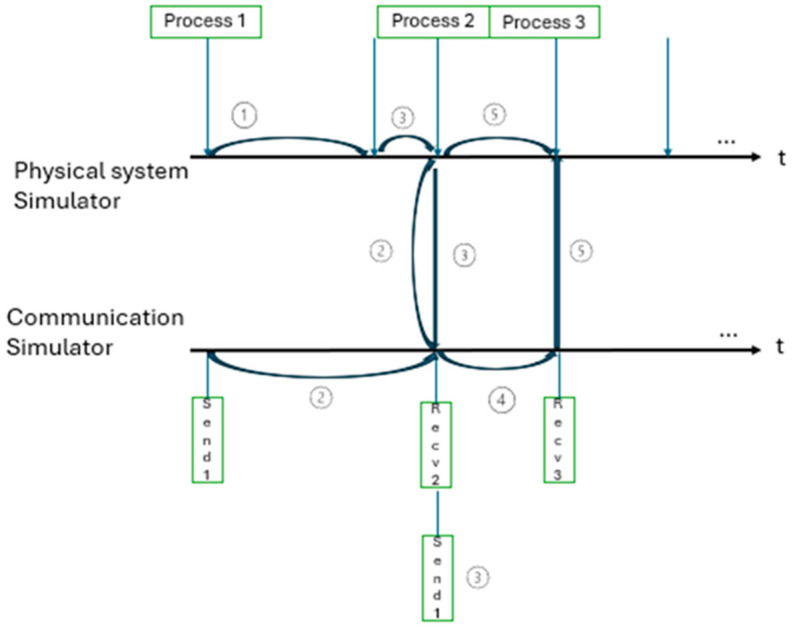
Demonstrative illustration of the variable-stepped method (after [6]).

Figure 3 shows the first transmitted message received earlier for the two previous simulators.

## 4. Implementation of the Co-Simulation Framework

### 4.1. Prototype Architecture

Three types of architecture were analyzed for the initial stage and for diversity as follows:

- Agent-based architecture models the behavior of several autonomous software agents that can interact with the simulation environment with high learning capacity, correcting the results in the evolution process.
- High-Level Architecture (HLA) [26] is a domain-independent reference through the integration characteristic of different simulators and the synchronization between them, and it has isolated and independent architecture components but is known as a run-time infrastructure.
- Hardware In the Loop (HIL) has data from hardware components integrated through co-simulators, interfaces, or support functions.

**Figure 4:**
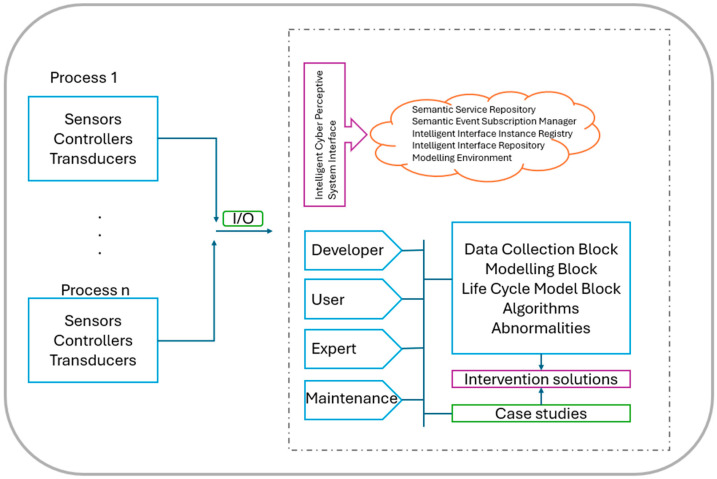
Conceptual architecture proposal of the CPPI.

The three aspects presented above (agent based, HLA, HIL) are quite compelling for the field of co-simulation. To integrate each concept, the platform design proposed in Figure 4 is presented, which integrates several functional components as follows:

- For data exchange, a perceptive cyber system;
- For storing and managing algorithms, a library;
- For control and parameterization, an intelligent interface;
- For predictive and proactive management, asset management;
- To mitigate critical situations, a component that uses machine learning and artificial intelligence;
- For the virtual rendering of the geal system, a digital twin (DT) component interconnected with the rest of the platform.

### 4.2. Design of the Cyber–Physical Perception Interface (CPPI)

There are four stages that form the procedure followed by the co-simulator. A preliminary step is the initialization stage for each simulator. In this way, the network simulator is prepared for the first event; then, the physical system specifies the sampling step, denoted by Δt, following the cycle below.

- In the first stage, it is assumed that the time of both simulators is t1, based on which the network simulator is commanded by the physical system simulator to advance the time after advancing its own time.
- The command was received from the physical system simulator, at which point the network simulator tries to advance the time to t1 + Δt for all iterations where there is no event in the chosen sampling step.
- If t1 and t1 + Δt in the interval, there is at least one event; then, the time of the network simulator advances until the time of the appearance of the oldest event, which is further denoted by t2. Furthermore, the physical system simulator is informed by the network simulator about the content of the event and the time t2.
- The state in the simulator is updated only after the time t2 is moved by the physical system simulator, thus becoming the simulator with the times at the same moment.
- The CPPI offers numerous advantages for integrated systems.
- Real-time Monitoring: The ability to collect and analyze data in real time allows for prompt detection of anomalies and quick intervention.
- Process Optimization: Advanced analyses and machine learning algorithms can identify opportunities for process optimization, reducing resource consumption and operational costs.
- Enhanced Security: Secure communication protocols and cybersecurity mechanisms ensure the integrity and confidentiality of transmitted data.
- Interoperability: The ability to integrate with various devices and systems allows the CPPI to operate efficiently in a heterogeneous environment.
- Interfacing with Analyzers and Sensors: The CPPI directly connects to distributed analyzers and sensors that collect essential data about the system’s operational parameters. These sensors monitor variables such as temperature, pressure, humidity, vibrations, and other critical environmental conditions. Analyzers, on the other hand, perform detailed analyses and provide precise information about the chemical composition of raw materials or the quality of the final product.
- Integration of PLCs: PLCs are fundamental components of automated systems, responsible for controlling and monitoring industrial equipment and processes. The CPPI interacts with these PLCs to receive and send commands, ensuring that all operations are carried out according to predefined specifications. This integration allows for real-time control and dynamic adjustment of processes, increasing efficiency and reducing the risk of errors or malfunctions.
- Communication Network: An essential component of the CPPI is the communication network that facilitates the exchange of information between all connected devices. This network uses robust and secure communication protocols to ensure the fast and reliable transfer of data. Technologies such as Industrial Ethernet, Modbus, Profibus, and OPC UA are often used to enable interconnection and interoperability between sensors, PLCs, and the CPPI. Additionally, the use of wireless networks and IoT (Internet of Things) technologies allows for greater flexibility and scalability of the system.

The CPPI plays a crucial role in managing and optimizing complex processes, and it is designed to integrate and coordinate signals from various devices, including analyzers, sensors, and Programmable Logic Controllers (PLCs), within an efficient and secure integrated system.

## 5. Case Studies

In order to validate the advantages of using the CPPI platform in applications that involve co-simulation, we chose two areas where there are already achievements with which we can compare our results.

The first is that of power systems based on Smart Grids, for which there is already an operational implementation carried out within the projects [27,28] in which holistic optimization of energy consumption for the entire product batch based on intelligent predictions was pursued. For this purpose, it was resorted to combining digital twin (DT) technology with the emerging IoT and AI achievements to support energy-aware production.

We applied the integrated predictive maintenance methods for real-time optimization of maintenance processes aimed at reducing costs and increasing productivity. In the DT virtual environment, the industrial process was simulated by a specialized software package, while separately, the platform retrieved data from the communication network through the CPPI.

Currently, we propose testing the first case study (CS1) using the CPPI platform for the dynamic study of the impact that a defect in the energy system can have on the communication system and vice versa.

The second field for which the other case study (CS2) is proposed is that of MCPS, under obvious development, which integrates the cyber world aspects and the real world with dynamic, fully flexible systems for decision making and other healthcare applications. In particular, CS2 is associated with a project that is currently underway, called SIMBIO [29], which aims to design, implement, and evaluate an in vitro Bio-medical Simulation Equipment (BSE) capable of evaluating the characteristics, dynamic behavior, and efficiency of a prosthetic implant prosthesis by capturing bio-signals using a cyber–physical interface.

### 5.1. Co-Simulation Scheme for Smart Grid (SG)-Based CPPSs

To evaluate the CPPS performance, the simulation method is necessary, and within the real physical energy network and the communication network, a collaborative simulation platform is proposed [30,31], with the possibility to choose between non-real co-simulation and real-time co-simulation. For the energy sector, the processes are quite dynamic, and co-simulation with real-time data must be chosen, implying that the simulation software can run in real-time, and the model must be divided into multiple pieces for parallel calculation. Commercial platforms that allow such co-simulations of power supply systems in real time, such as OPAL-RT [32] or Matlab [33], show relatively high complexity, but they can be adjusted from the characteristics and initialization functionalities. In this work, the Matlab development environment was used to perform co-simulation by setting the time factor of both simulators to the same value, with physical time synchronization being possible.

**Figure 5:**
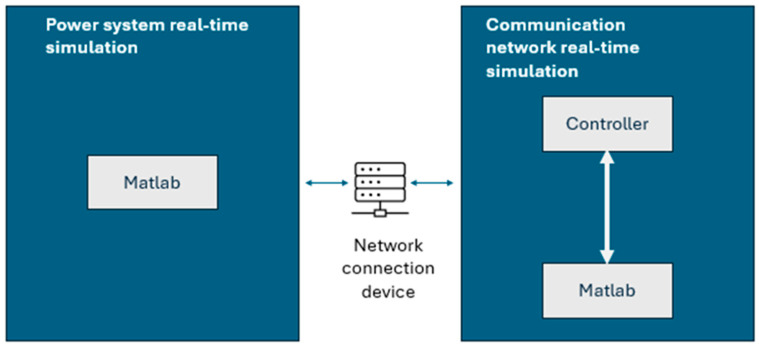
Structure of real-time co-simulation using Matlab.

Figure 5 illustrates the structure of a real-time co-simulation using Matlab. This involves integrating multiple hardware and software components to run real-time simulations. The system is composed of a Matlab/Simulink interface, which communicates with the hardware platform via communication protocols, such as TCP/IP.

Case study (CS1), which is used to correctly simulate the interaction between the actual physical power grid and communication network, is present in the ‘Smart digital Solution for Local Green Energy Management’ (SMARTGEM) [28] project; the project team successfully implemented a secure communication system between two geographically separated SCADA (Supervisory Control and Data Acquisition) locations using advanced network technologies and standardized communication protocols. The main objective of the project was to ensure reliable and secure data transfer between the monitoring and control equipment in the two locations, hereinafter referred to as Location A (Constanta city) and Location B (Capital Bucharest).

Configuring a VPN for Secure Connectivity: To ensure a secure communication channel, we opted to use a VPN (Virtual Private Network). The process involved the following essential steps:

- VPN Server Setup at Location A: We installed and configured a VPN server on the main router at Location A. This VPN server was responsible for handling secure connections from Location B. Encryption was set up to protect the data being transmitted, ensuring that they cannot be intercepted or modified during transfer;
- Configuring the VPN Client at Location B: On the router at Location B, we configured a VPN client to automatically connect to the VPN server at Location A. This connection has been tested for stability and security;
- After establishing the connection, I verified that all the devices in Location B could communicate with those in Location A using internal IP addresses as if they were part of the same local network;
- To allow the specific traffic required for SCADA operations, we made the following configurations on routers and local networks;
- Port Forwarding: At Site A, we configured port forwarding on the router to allow access to port 502, which is used by the Modbus TCP/IP protocol, from the SCADA devices at Site B. This ensured that all Modbus data requests could be transmitted and received correctly;
- Firewall Rules: We defined strict firewall rules on both Site A and Site B to allow only authorized traffic on required ports. These rules also included checking the IP address of devices at the other location to prevent unauthorized access;
- NAT (Network Address Translation): We used NAT to map internal IP addresses to public IP addresses, facilitating communication between devices on private networks and the Internet without compromising security.

To ensure efficient and accurate communication between SCADA equipment, the following appropriate Modbus functions have been implemented:

- Read Function (0x03): We configured the Modbus client at Site B to send read requests to the Modbus server at Site A, specifying the necessary register addresses. These applications enabled real-time monitoring of critical parameters from remote equipment;
- Write Function (0x06): We configured the Modbus client at Site B to send write commands to the Modbus server at Site A, allowing updates of control settings and parameters. These commands have been validated to ensure data integrity.

**Figure 6:**
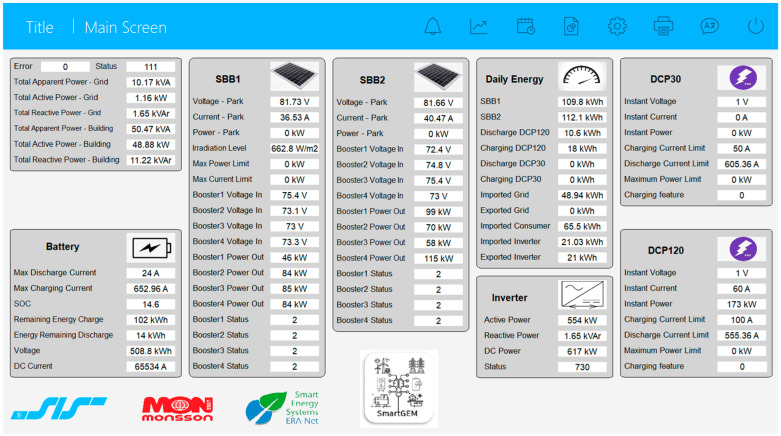
Implementation of secure communication between SCADA locations.

The screen in Figure 6 is developed in the PcVue environment [34], and it is a flexible SCADA solution for the supervision of industrial processes, services, and infrastructures. The SCADA application starts automatically when the computer is turned on. When starting the application, the general screen automatically opens, where the key performance indicators in the system are presented. This screen is an information management tool used to track, analyze, and display data from the battery, SBBs, DCPs, and the inverter.

The values shown in these screens are collected via the Modbus TCP protocol. The main parameters monitored are status, voltage, current, active and reactive powers, load and discharge limits, methodological data, and daily energies calculated in the PLC, as well as values of the instantaneous powers provided by the park and network and the power from consumers or the input in the network.

PcVue offers flexibility in using multiple history servers and HDS to log data to an SQL Server database, with various possible configurations. Configuration 1 involves multiple active servers and multiple SQL servers, with each server logging into its own dedicated SQL Server and with the advantage of using the same project on all servers, but there are high costs due to multiple SQL Server licenses. Configuration 2 involves multiple active servers logging into the same SQL Server database but in different tables, offering lower costs but requiring different projects for each server. Configuration 3 uses a single active server logging to the same table of the same SQL Server database, and it is the most cost-effective and allows the same project to be used on all servers without requiring a replication mechanism.

Exporting data from archives and transferring them from HDS servers involves specific steps to access, select, transfer, and convert data into accessible formats, such as CSV or Excel. Subsequently, statistical analysis of these data by calculating the mean, maximum/minimum limit, and standard deviation allows for a deeper understanding of the information and support for informed decisions.

MATLAB accesses data from Excel files using specialized functions that make it easy to read and write data. The ‘readtable’ function is often used to import data into a MATLAB table format, allowing for easy manipulation. The ‘xlsread’ function also allows data to be read from a specific sheet of an Excel file, returning the data to a MATLAB array or data structure. To export data to Excel, the ‘writetable’ function can be used to write the data from a MATLAB table to an Excel file, and ‘xlswrite’ allows writing the MATLAB matrix directly to an Excel spreadsheet. These functions simplify the transfer and manipulation of data between MATLAB and Excel, facilitating complex analysis and visualizations.

**Figure 7:**
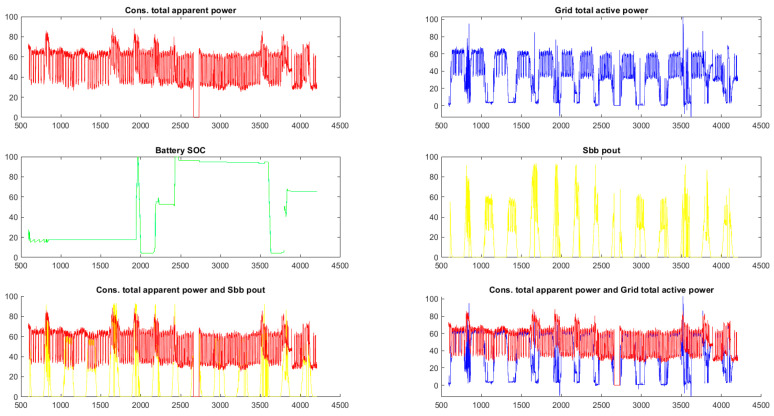
Consumer total apparent power, grid total active power, battery state of charge, SBB power out graphs for March 2022.

The structure of real-time co-simulation using Matlab in a power system is shown in Figure 7. Data centralization from March 2022 and data exported from the SCADA data server were used. The reading of these data was performed through functions in Matlab, such as ‘Data_Mar = xlsread(‘March2022.xls’);’, which allows reading the data and making graphs or other statistics. The state of charge (SOC) indicates the level of charge that the battery has within a photovoltaic system, and it is is divided into four String Box Busters (SBBs) and has an installed power of approximately 120 kW. It can be observed that the production (represented in yellow) is clearly lower than the consumption (in red), so the photovoltaic system is undersized. But, this can be given by the limited area of the location, the investment cost at the current moment, a division of the renewable investment by years, a thought of dividing the energy mix that the co-tenant uses, etc.

The physical system that produced cyber disturbances created different scenarios after running the cyber physics simulation platform. The scenarios were taken one by one, and the resulting parameters were analyzed, such as voltage and loss of active power.

The present work proposes a solution that provides a versatile cyber–physical co-simulation that is able to meet the requirements of modularity and integration in various applications. This article presents a real microgrid case study, demonstrating the use of co-simulation in a network configured between two locations that transmit data to each other in real time, thus facilitating communication between the simulator and the controller. The simulation involved the use of two algorithms introduced both in the simulator and the controller from the real microgrid process. The initialization conditions were almost simultaneous, as the data packets were initially received by the microgrid controller and then archived in the SCADA application and sent to Matlab for processing. Communication was maintained and performance was monitored throughout the simulation. The results showed that although simulations tend to be faster for tests and rapid iterations, real processes are essential for final validation and to ensure the correct functioning of algorithms under real conditions. In the case study, it was observed that the co-simulator used responded faster than the results obtained from the physical network controller.

### 5.2. Co-Simulation Scheme for an Esophageal Phantom

This case study aims to analyze and evaluate the characteristics, the dynamical behavior, and the efficiency of a personalized implantable active pharyngo-esophageal prosthesis, which is defined as a phantom (a dedicated in vitro simulation equipment) for patients who received an implant with this prosthesis. The prosthesis is intended to replace the excised pharynx and esophagus during a total or partial circular pharyngo-laryngectomy. The research in this project does not focus on prosthesis design (for which there is already an approved patent) but a simulator of it, which allows in vitro tests to emulate in vivo peristaltic movements.

**Figure 8:**
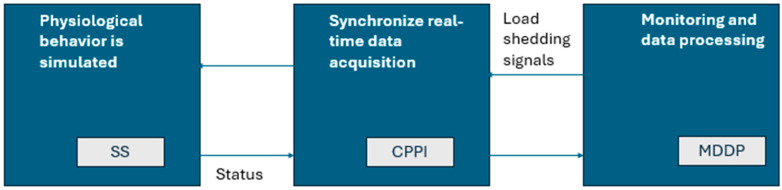
Semi-physical co-simulation scheme.

The simulation equipment is presented in Figure 8 and consists of three main components: a Smart Simulator (SS) that allows the reproduction of the movements controlled by artificial muscles, which is equipped with appropriate sensors; a cyber–physical perception interface (CPPI) to pick up physiological signals; and a monitoring and data processing platform (MDPP) provided by bio-medical sensors. The equipment allows for a study on the basis of personalized scenarios in the sense that they are dedicated to a specific patient. To achieve these objectives, original algorithms will be developed to synchronize real-time data acquisition through the CPPI and to analyze the time series of medical data through MDPP. This time, co-simulation refers to the agreement of a physical simulator (PS), which provides contextual data based on the HIL principle, and a software simulator, which can be structured as a DT. However, in this situation, the problem of real-time co-simulation no longer arises because the data analysis takes place offline. This is why a scheme called semi-physical simulation [7], presented in Figure 8, was chosen for the prototype of the co-simulation framework.

Through a software simulation of the replaced medical equipment and the obtained behavior, the results of a semi-physical simulation are reproduced, and they are used in different research topics. In this way, the semi-physical simulation renders the result after the introduction of the real object with simulation data, and the accuracy of the simulation will increase using a real-time interface with a response in both directions, both to the simulator and the physical representation.

Simulations with real-time data and the concept of a digital twin [35] are revolutionizing the healthcare sector, especially in the development and use of prostheses for esophageal cancer. By integrating real-time data from devices and sensors, digital twins create an accurate virtual replica of the patient or a specific organ [36], such as the esophagus. These digital models allow doctors and researchers to monitor the patient’s condition in real time, simulate different treatment scenarios, and adjust prostheses in a personalized way. In the case of esophageal cancer, the use of a digital twin can optimize the design and function of esophageal prostheses, ensuring a perfect fit and significantly improving clinical outcomes by anticipating possible complications and personalizing therapeutic interventions. This advanced approach [37] offers the potential to transform cancer treatments and improve patients’ quality of life.

## 6. Conclusions

In this paper, Cyber–Physical Production Systems (CPPS) have been discussed as an innovative approach that integrates the co-simulation of physical systems, such as medical and energy systems, with advanced communication systems. This integration allows for a more realistic and accurate simulation of the interactions between physical and cyber components. Medical systems, for example, can benefit from simulations that accurately replicate the physiological behavior of patients, while energy systems can accurately simulate energy flows and the behavior of smart networks. Co-simulation also facilitates better coordination and synchronization between the various subsystems, resulting in overall performance optimization and better resource management. Therefore, CPPS represents a viable and effective solution for improving the interoperability and efficiency of complex systems while ensuring better adaptation to current needs and requirements.

In conclusion, the present work demonstrates the efficiency and versatility of the proposed solution for cyber–physical co-simulation, highlighting its ability to meet the requirements of modularity and integration in various applications. The microgrid case study validated the use of this solution by implementing a network between two locations that transmit data in real time, facilitating effective communication between the simulator and the controller. The experimental results confirmed that although simulations allow for faster tests and iterations, real processes are crucial for the final validation of the algorithms and for ensuring correct operation under authentic conditions. The observation that the co-simulator responded faster than the physical network controller suggests that the proposed solution not only improves the efficiency of simulations but also provides a solid foundation for the application and development of future cyber–physical technologies.

In the future, the authors will focus on the application and extension of the working method demonstrated in the real case study, which formed the basis for the development of the research pillars. This approach will be used as the foundation for the second case study, thus defining the main direction of the team’s research. In addition, the authors plan to analyze a larger number of algorithms, including several that are currently under research, such as analyzing the influence of variations in the geometry of the prosthesis, evaluating the tissue response to the implant due to inflammation and foreign body reaction, detecting and predicting behavioral abnormalities by analyzing historical data collected over a long period of time, predictive algorithms for detecting abnormalities in medical data time series, and exploring strategies in the multimodal treatment of esophageal cancer. This additional research will contribute to deepening the understanding and refinement of cyber–physical co-simulation methodologies, thus supporting the advancement of the field.

## Data Availability

No new data were created or analyzed in this study. Data sharing is not applicable to this article.
